# The Evidence for the Pharmaceutical Strengthening of Attachment: What, *Precisely*, Would Love Drugs Enhance?

**DOI:** 10.1017/S0963180122000196

**Published:** 2022-10

**Authors:** Peter N. Herissone-Kelly

**Affiliations:** School of Humanities, Language and Global Studies, University of Central Lancashire, Preston PR1 2HE, UK

**Keywords:** love drugs, Julian Savulescu, oxytocin, vasopressin, attachment

## Abstract

In recent decades, scientists have begun to identify the brain processes and neurochemicals associated with the different stages of love, including the all-important stage of attachment. Experimental findings—readily seized upon by those bioethicists who want to urge that we sometimes have good reason pharmaceutically to enhance flagging relationships—are presented as demonstrating that attachment is regulated and strengthened by the neuropeptides oxytocin and vasopressin. I shall argue, however, that often what the experimental data in fact show is only that exogenous administration of such chemicals can control and intensify the trappings of attachment, not attachment itself. That this is sometimes overlooked by both scientists and ethicists, is due to attachment being miscategorised as a set of feelings or a drive, rather than as a disposition to think about, feel toward, and behave toward its object in certain distinctive ways.

## Introduction

In recent decades, scientists have begun to identify the brain processes and neurochemicals associated with the different stages of love. The biological anthropologist Helen Fisher famously identifies three such stages: lust, romantic attraction (which she sometimes calls “romantic love”^1^), and attachment. While lust drives us to seek out any appropriate partner for mating, attraction narrows our attention down to a particular individual. Attachment represents the formation and maintenance of a stable pair-bond, enabling cooperation in the raising of offspring. Each of these stages, neurological research shows, is associated with its own brain system—indeed, somewhat controversially from a philosophical perspective, Fisher *identifies* each stage with a brain system—and thus with its own suite of neurotransmitters or hormones. For example, testosterone is implicated in lust; norepinephrine and dopamine in attraction; and oxytocin and vasopressin in attachment. These findings open up a dizzying prospect. It is hypothesized that, if we were exogenously to administer these endogenously occurring substances, we would perhaps be able deliberately to manipulate, or at least to influence, the hitherto unpredictable and unbiddable stages of love in line with our values and preferences. What is more, the claim goes, that hypothesis is to some extent borne out by experimental findings. I will have more to say about some of those findings later.

Amongst bioethicists, the chief enthusiasts for the use of so-called “love drugs” (once they have been developed and adequately tested) in this way is a small group of coauthors clustering around the Oxford moral philosopher Julian Savulescu—a group that prominently includes Brian Earp and Anders Sandberg.^2^ These authors maintain not only that there is insufficient cause for ethical queasiness about the carefully regulated, suitably consensual employment of (certain) love drugs, but that quite often couples will have good reason to make use of them. To be more precise, and importantly given the focus of the current paper, what they primarily advocate is the use of what we might call “attachment enhancers”—in the shape, for example, of deliberately administered oxytocin—ideally in combination with traditional relationship therapy, in order to help bolster flagging relationships (they are cautious and unenthusiastic about the use of drugs to initiate new relationships^3^). They do so against a background of the sorts of scientific data reported by the likes of Fisher, which purportedly show that attachment is neurochemically produced and sustained, and could therefore in principle be pharmaceutically enhanced.

A lot depends, then, on whether the use of substances such as oxytocin have adequately been shown through experiment to cause, strengthen, or deepen human romantic attachment. Whether that is the case, I want to urge, depends in part on what sort of phenomenon attachment is. If we are unclear about the ontological category into which it fits, we may take attachment to have been enhanced through, say, the exogenous administration of oxytocin or vasopressin, when in reality it is perhaps something else, something merely related to attachment, that has been shown to be boosted. Those championing the use of love drugs tend to adopt wholesale an account of the nature of attachment from the scientific literature: one that conceptualizes it as, variously, a drive, appetite, feeling, or sensation. But in fact attachment is none of these things. Once we correct the error and get clearer about what attachment is, we will see that at least some of the experimental data on which enthusiasts draw show at most that the *trappings of attachment* can be enhanced through the use of love drugs. Those data do not show that *attachment itself* can be pharmaceutically enhanced.

In the section “What Sort of Phenomenon Is Attachment?” I will argue against the claim that romantic attachment is properly categorized as a drive or appetite. I will then go on in the section “Pharmaceutical Strengthening of Attachment: Assessing the Putative Evidence” to contend that while the sort of experimental data that proponents of love drugs appeal to may support the claim that feelings of attachment and attachment-related behaviors are strengthened through the administration of oxytocin or vasopressin, they do not always thereby support the claim that attachment itself is strengthened.

## What Sort of Phenomenon Is Attachment?

In her popular science book *Why We Love*, Fisher categorizes all three stages of love—lust, attraction, and attachment—as drives. However, her arguments for this taxonomical move are focussed only on the second of those stages, which in the book she contentiously labels “romantic love.”^4^ What is more, the argument that she apparently takes to be paramount is, to say the least, suspect. She writes “Very important [*sic*], all of the basic drives are associated with elevated levels of central dopamine. So is romantic love.”^5^ We are clearly invited here to draw the conclusion that romantic attraction is therefore a drive.

Let us take a closer look at this argument, and at the assumptions at work in it. Before we were able to notice that all the basic drives have the tie to the action of dopamine that Fisher mentions, we must first have been able to identify them as drives independently of that chemical association. We consequently must have done this on some grounds other than the existence of the association (indeed, as we shall see shortly, Fisher herself sets out some nonchemical criteria for something’s counting as a drive). That done, we must have noted that everything we independently count as a basic drive is associated with elevated levels of central dopamine. It is only with this established that Fisher is able to reason that romantic attraction also is a drive, just because it too is associated with elevated levels of central dopamine.

Unfortunately, this argument commits the formal fallacy of affirming the consequent. Its form is this:

1. For all *x*, if *x* is a drive, then *x* is associated with elevated levels of central dopamine.
2. Attraction is associated with elevated levels of central dopamine.
3. Therefore, attraction is a drive.

If it is not clear that this argument is invalid, compare the following piece of reasoning, which displays exactly the same form:

1. For all *x*, if *x* is an apple, then *x* is a fruit.
2. This kumquat is a fruit.
3. Therefore, this kumquat is an apple.

Fisher may retort that her argument is not intended to be deductively valid, but is, in keeping with the bulk of scientific reasoning, meant merely to be inductively strong. It does not, that is, conclusively show that attraction *must* be a drive; instead, it suggests that there is a high probability that it is. Taken by itself, it is not obvious that it does even that much, any more than the parallel argument shows that a given kumquat is likely to be an apple.

Since Fisher’s argument is unable to show that attraction is a drive, it would be equally ineffectual as an attempt to prove that attachment is a drive. Nonetheless, Fisher unequivocally categorizes attachment in that way (although she neglects to mention that there is a link between attachment and dopamine, other authors make it clear that there is^6^):

Romantic love is deeply entwined with two other mating drives: *lust*—the craving for sexual gratification; and *attachment*—the feelings of calm, security, and union with a long-term partner.^7^

Two things are noteworthy here in addition to the identification of attachment as a drive. First, while pretheoretically it would seem plausible to count lust as a drive, I suspect many people would intuitively be disinclined to characterize attachment in the same way. Secondly, it is interesting that in addition to describing attachment as a drive, Fisher identifies it with a set of *feelings.* This is not necessarily a slip. Hunger is quite legitimately counted as both a feeling and a drive; indeed, we may think that the feeling of hunger *just is* the motivating factor—the drive—that prompts us to seek out food. Nonetheless, it is difficult to say what action “feelings of calm, security, and union” are supposed to propel us toward. The most favorable candidate might simply be continued contact with the person who induces those feelings. In the main, however, the feelings listed seem more correctly described as the result of such contact, rather than the motivation for it. Given that there are these considerations pulling in the opposite direction of our counting attachment as a drive, we need strong reasons to accept Fisher’s contention.

As mentioned above, Fisher has a set of reasons *R* for classifying a given phenomenon *P* as a drive, such that *R* is quite distinct from *P*’s association with elevated levels of central dopamine. Indeed, it *must* be the case that she can point to such a set. As I have said, her central argument requires that phenomena *independently identified as drives* be shown subsequently to be associated with heightened dopamine levels, in order to arrive at the crucial premise that a link exists between all major drives on the one hand, and elevated levels of dopamine on the other. That argument, consequently, cannot even get off the ground without the aid of *R.*

What, then, are the reasons that make up *R*? Romantic attraction, Fisher tells us, has the following features, which constitute reasons to categorize it as a drive:

1. it is, unlike emotions, which tend to come and go, tenacious;
2. it is focussed on a specific reward, just as a drive like hunger is;
3. it is, unlike emotions, not associated with any particular facial expression;
4. and it is, more so than emotions, very difficult to control.^8^

Once she has given us this list, all may not be lost for Fisher’s contention, despite the failure of her central argument. That is, if these really are reasons to regard a phenomenon *P* as a drive—if, to be more precise, they are factors that are necessary and sufficient for *P*’s being a drive—then their being possessed by romantic attraction establishes that such attraction is indeed a drive, without the need to appeal to any association with heightened dopamine levels. What is more, if attachment has those features as well, then it too can quite correctly be counted as a drive.

However, it is possible to question whether the list is truly able to show that either attraction or attachment is a drive. First, it will not have escaped notice that Fisher appears to be working with a rather simplistic binary choice: attraction and/or attachment are either drives, or they are emotions; they are too dissimilar to paradigm cases of emotions to be classified in that way; therefore, they must be drives. But why suppose that those two categories exhaust the possibilities? Might not these romantic phenomena be something else, such as—plausibly, as I shall suggest shortly in the case of attachment—dispositions to feel, think, and act in certain ways? Secondly, on closer examination the similarities between these romantic phenomena on the one hand and paradigm cases of drives on the other are not actually as great as Fisher suggests.

This latter fact is highlighted in the work of Andrew McGee,^9^ although McGee’s target is the output of Savulescu and colleagues, rather than (at any rate, directly) that of Fisher. That being the case, he uses Savulescu et al.’s terminology of “appetites”^10^ rather than Fisher’s talk of “drives,” though it seems that these two expressions both pick out the same type of phenomenon. In addition, he discusses the issue of whether love itself, rather than any of its phases, is an appetite/drive. Nonetheless, it seems that what he has to say about love will apply equally well to the case of romantic attachment.

According to McGee, love (and, we can add, attachment) has properties that distinguish it from paradigm cases of appetites/drives. Whereas drives such as hunger lack specific objects (one has hunger simply for food, rather than for a certain sort of food), attachment is focussed on a particular person or persons. Satisfying an appetite—for sex, say, or again for food—leads to temporary satiation. In the case of attachment, this does not happen: being close to the object of one’s attachment does not result in that attachment temporarily abating, only to build up again after a period of absence from him, her, or them. Relatedly, while appetites or drives are recurrent, attachment is constant.^11^

So, whereas we might, it seems to me, correctly label lust as a drive (it can be satiated; it recurs; and so on), I think it would be wrong to categorize attachment—and indeed attraction—in the same way. What sort of phenomenon, then, is romantic attachment? What type of thing would we be trying to boost or strengthen through the use of attachment enhancers?

It seems uncontroversial to say that a person *A* is attached to another person *B*, if and only if *A* is disposed to think, feel, and behave in certain characteristic ways with regard to *B.* For attachment to be in place, that disposition must be more or less settled: *A*’s display of a single island of affectionate behavior in a sea of indifference toward *B* would not, or at any rate should not, lead us to count *A* as attached to *B.* The question then is whether attachment ought to be thought of as something that underlies and explains this more or less settled disposition, or whether it ought to be identified with the disposition itself. Whichever of these options is embraced, the *constancy* of attachment—as mentioned by McGee—is appropriately acknowledged: either way, attachment is not something episodic that vanishes whenever its manifestations are absent. However, it is not clear what would be gained by our subscribing to the former option over the latter. Doing so would amount to our identifying attachment as the categorical ground of the disposition to behave in certain ways. There is no reason to prefer this classification to a simpler one that characterizes attachment as that disposition itself.

If this is the correct understanding of attachment—and I think it is—then a successful attachment-enhancing drug would serve to strengthen or further embed a disposition to think of, feel about, and behave toward another person in certain characteristic and identifiable ways. It would, so to speak, make that disposition *more of a disposition.* That is, it would render it more settled, more resilient, less vulnerable to the vicissitudes of external and internal circumstances.

## Pharmaceutical Strengthening of Attachment: Assessing the Putative Evidence

A range of experimental data is cited in the scientific and bioethical literature in support of the claim that exogenous administration of oxytocin or vasopressin can strengthen or intensify attachment. Appeal is often made here to animal models. For example, a paper by Larry J. Young, Zuoxin Wang, and Thomas R. Insel tells us that endogenous oxytocin and vasopressin, released during mating, have been shown to be implicated in the formation of monogamous pair-bonds in prairie voles. What is more, prairie voles to whom those substances are exogenously administered form the same sorts of bonds even in the absence of mating. In both cases, it seems entirely legitimate to characterize the disposition engendered as attachment, of the sort that aids cooperative parenting:

Pair-bonded males prefer the company of the mate and exhibit ‘selective’ aggression towards other members of the species. The breeding pair nests together: both parents provide extensive, prolonged parental care, and the offspring remain in the parental nest for several weeks beyond weaning.^12^

It is hypothesized that, given the conservative nature of evolution, oxytocin and vasopressin will have a similar effect in humans, whether endogenously produced or exogenously administered. And again, a range of putative evidence—readily seized upon by the proponents of love drugs—is held to confirm that hypothesis. That is not to say that no differences at all are posited between the effect of the relevant neuropeptides on prairie voles, say, and the manner in which they affect human beings. The results for prairie voles are portrayed as more or less automatic. Not so for humans—a fact that those claiming we have good reason to take love drugs repeatedly emphasize, seemingly to allay any worries that their use would give us an undesirable level of control over our romantic lives. Earp, Savulescu, and Sandberg, for example, write that a substance such as oxytocin.

… would not work to create love ‘magically,’ of course, but it might certainly help it along by acting on the underlying substrates of attachment, or by promoting more empathic states of mind.^13^

In this quotation, we are presented with two possible accounts of the action of a love drug. Oxytocin, say, might work directly on the neural correlates of attachment (what the authors call, somewhat pleonastically, attachment’s “underlying substrates”), or it may produce some other phenomenon (“more empathic states of mind”) that will provide fertile ground for the development of attachment, without compelling that development. Elsewhere, I call the former hypothesized mode of action “the productive account,” and the latter “the facilitative account.”^14^

There would appear to be two possible interpretations of the productive account, both of which would fit with Savulescu et al.’s occasional echoing of the scientific literature’s talk of neurochemicals *regulating* or *modulating* the various stages of love.^15^ The thought could be either (1) that the action of oxytocin directly produces or strengthens attachment, but that attachment is not sufficient for love; or (2) that the action of oxytocin can directly produce attachment, but only when numerous other enabling conditions cooperate. Whichever of (1) or (2) is correct, and indeed whether the productive or the facilitative account is deemed accurate, the claim is that whenever exogenously administered or endogenously produced oxytocin works, attachment or the strengthening of attachment is the (direct or indirect) outcome. Let us call this broad claim (A), for “attachment.”

The problem, however, is that some of the empirical evidence appealed to in support of (A) does not in fact support (A) at all. Instead, it supports a wholly distinct claim—call it (A*)—that when exogenously administered or endogenously produced oxytocin works, the occurrence or strengthening of *certain phenomena associated with but not identical to* attachment is the (direct or indirect) outcome. In order to show that this is the case, I want to consider some of the putative evidence for (A) that is presented in the scientific literature, and that is cited by the proponents of love drugs.

First, a study outlined by Wudarczyk, Earp, Guastella, and Savulescu shows that brain areas containing receptors for oxytocin, vasopressin, and dopamine are more active when subjects look at photographs of their romantic partners, than when they look at images of platonic acquaintances, suggesting a link between the activity of these areas and attachment. These echo similar results in studies that expose securely attached mothers to photographs of their own children on the one hand, and of unrelated children on the other.^16^

Secondly, the same authors cite the following set of experiments on the effects of an oxytocin nasal spray:

[I]n one study, oxytocin-primed male participants who were in a committed heterosexual relationship—but not single males—kept themselves at a significantly greater distance from an attractive female experimenter during an initial personal encounter. Such males also showed a decreased reflexive approach response when exposed to erotic images of beautiful women as measured by computerized approach-avoidance paradigm. These findings suggest that intranasal oxytocin may help to promote fidelity toward one’s current partner by contributing to the ongoing maintenance of an existing pair bond.^17^

The most natural interpretation of the first study’s finding, I would suggest, is not that the action of oxytocin is responsible for attachment, but rather that attachment is responsible for the action of oxytocin. That is, the best explanation of why regions of the brain dense in oxytocin, vasopressin, and dopamine receptors light up when we are confronted with images of loved ones, in a way that they do not when we are faced with pictures of people who are for us more “neutral,” might seem to be that we are attached to the former group and not to the latter. But in that case, the activity of those areas of the brain would be the result of attachment, or a correlate of one of its manifestations, rather than the biochemical ground of it.

Now, one reason we may not opt for this “natural” interpretation would be if we were mistakenly to identify attachment, not with a disposition to feel, think, or behave in certain characteristic ways, but with the sorts of phenomena that can count as individual manifestations of that disposition. I mentioned earlier, for example, that Fisher identifies attachment with a set of feelings. Her doing so is apparently not a slip, since she does it repeatedly. Discussing attachment in *Why We Love*, in the space of a handful of pages she makes the following claims (all emphases are mine):

Only recently … have researchers begun to understand which brain chemicals produce this *feeling of fusion* with a long-term mate …. [T]hese hormones generate the *sensation of union* with a sweetheart ….^18^

[T]he *feeling of attachment* must be a common *sensation* among all birds and mammals, because it is associated not only with vasopressin but also with oxytocin—a related hormone that is ubiquitous in nature.^19^

[M]any now believe that oxytocin is … involved in the *feelings* of adult male–female attachment.^20^

You have undoubtedly *felt* the power of these two ‘satisfaction hormones,’ as vasopressin and oxytocin are sometimes called …. These ‘cuddle chemicals’ undoubtedly contribute to that sense of fusion, closeness, and attachment you can feel after sweet sex with a beloved.^21^

Under some circumstances, dopamine and norepinephrine can stimulate the release of oxytocin and vasopressin—and contribute to one’s growing *feelings of attachment.* ^22^

[R]omantic love gradually transforms into *feelings of deep attachment.* ^23^

If attachment is a feeling (and thereby perhaps a drive, as we saw in the last section), then we can understand how it might arise in an occasional manner, and be correlated with episodic increased activity in brain regions rich in oxytocin and vasopressin receptors. However, we would still be owed an explanation of why the feeling arises in response to photographs of a partner, but not to images of platonic acquaintances. The explanation could not take the form, “Because the subject is romantically attached to the partner and not to the acquaintances,” because on Fisher’s account the attachment would *be* the feelings, and therefore not a contender for the factor that explains their existence.

What is more, even though Fisher uses the expression “feelings of attachment” as if it were coextensive with “attachment,” this cannot be how things stand. There are two candidates for the sort of thing we might call a “feeling of attachment,” and neither of them is identical with attachment. So if, as the “photograph” experiment might seem to suggest, it is a feeling of attachment that is associated with and produced or strengthened by the action of vasopressin and oxytocin, then that is quite a different state of affairs from attachment’s being produced or strengthened by those neurochemicals. It is the difference between (A*) being true, and (A) being true.

First, the expression “a feeling of attachment” might pick out a feeling that one is attached. If one is accurately to feel that one is attached, one’s being attached must be a separate, conceptually prior state of affairs to the having of the feeling. This is because such a feeling belongs to a certain distinctive class. It is a feeling that one is in a state *S*, such that being in *S* transcends one’s feeling that one is in *S.* Another member of this class is the feeling that one is privileged. To have a feeling of that sort is importantly different from, say, feeling dizzy. One is dizzy for just so long as one feels dizzy; one feels dizzy for just so long as one is dizzy. But to feel privileged is to feel that one is enduringly in a state that outstrips one’s awareness that one is in it. It is, if you like, an episodic impression that one is nonepisodically privileged; the state of being privileged does not diminish when the feeling that one is in it fades. The privileged remain so even when in dreamless sleep, and thus entirely insensate. Exactly parallel things can be said about the feeling that one is attached. Assuming that the participants in the nasal-spray study felt attached at the moment they placed a substantial distance between themselves and the experimenter, they were able accurately to feel that way, just because they were already attached before they felt it. That being the case, feeling that one is attached is not the same thing as being attached.

The second possible referent of the expression “a feeling of attachment,” is any one of a set of feelings that a person is disposed to have when she is attached (a set composed of feelings such as affection, warmth, respect, concern for another’s well-being, and so on). To explain: to be attached to someone, as we established in the previous section, is to be disposed to think about them, feel about them, and behave toward them in a characteristic set of ways over an extended period of time. The possession of that disposition, of course, does not require that the relevant sorts of thinking, feeling, and behaving be present at every instant. Again, one can properly be counted as attached to a partner even when unconscious, or when one’s mind is wholly occupied by things that have no connection at all to that partner. I still count as attached to my partner when I am struggling to understand a complex proof in formal logic, for example, and the difficulty of that task crowds out everything else.

Let us use the label “attachment-related feelings” for the feelings that one is disposed to have when one is attached. Any particular attachment-related feeling *F* is something distinct from the disposition to have *F* and other feelings like it. That being the case, no attachment-related feeling or set of attachment-related feelings is identical to one’s attachment. What we have established, then, is this: *pace* Fisher, attachment is something distinct from a feeling of attachment, regardless of whether that feeling is a feeling that one is attached, or an attachment-related feeling. In addition, the “photograph” experiments seem to show that the action of oxytocin and vasopressin is something episodic, perhaps associated with or productive of attachment-related feelings. That being the case, the experiments do not demonstrate that the action of oxytocin and vasopressin is productive of attachment itself.

The findings of the experiments with an oxytocin nasal spray fare no better, if what they are intended to show is that administration of the spray strengthens attachment. Is what is strengthened by the action of oxytocin what we can properly call “attachment”: that is, the settled disposition to think, feel and act toward a partner in particular ways (including the having of attachment-related feelings)? Or is what is strengthened something distinct from this: namely, a feeling that one is attached? Or, finally, is it the set, or some subset, of attachment-related feelings?

Let us suppose, first of all, that the nasally administered infusion as a matter of fact *does* directly strengthen attachment proper: the settled disposition to think about, feel about, and behave toward a partner in certain characteristic ways. Experimentally determining that attachment has been strengthened is no easy task, and it is not clear that the nasal-spray experiment achieves it. A strengthening of the settled disposition is not necessarily a matter of an intensification of either feelings that one is attached or attachment-related feelings. Rather, it is primarily a matter of the disposition becoming *more settled*: that is, more resilient, more entrenched, and longer-lasting than it otherwise would have been. But to claim that this has happened is to state a series of counterfactuals, and establishing the truth of such counterfactuals is notoriously difficult: how can we determine, in a case such as the nasal-spray experiment, that a participant’s disposition is now more resilient than it otherwise would have been? One problem is that we will be given no clue at all about this in the short-term—resilience is something that can only be measured over a significant time-span. And even then, the judgment that the resilience of the disposition has increased involves more than the judgment that it *has* lasted a long time: in addition it includes the belief that it *will continue* to last, or would also have lasted in counterfactual situations in which greater obstacles had been placed in its way. How are such beliefs to be justified?

Now suppose that attachment-related feelings are intensified following administration of the nasal spray, and that these feelings motivate the subject to maintain a greater distance than he otherwise would have done from the female researcher. This outcome, it should be noted, does not entail that attachment itself has been strengthened—it would be perfectly possible for attachment-related feelings such as affection to become more marked, and yet for the resilience and the longevity of the relationship not to have been improved. The disposition itself need not have become more embedded, just because the feelings its subject is disposed to have are now more powerful.

Finally, suppose that what is strengthened by the administration of oxytocin is neither attachment itself, nor attachment-related feelings, but the feeling that one is attached. That is, under the influence of exogenous oxytocin, the participant feels more attached than he did before the experiment started, and this explains the distance that he places between himself and the female experimenter. Again, we need to note that feelings that one is attached and attachment itself are distinct. The participant’s *feeling* more attached does not entail that he in fact *is* more attached. The feeling may be illusory: no strengthening of his settled disposition may have occurred.

Let me summarize what has just been claimed. It is unclear from the nasal-spray experiment just what is being strengthened. The study does not supply any evidence that attachment itself is directly magnified, just because, under the parameters of the experiment, an increase in attachment would not be detectable. All that could be detected would be, subjectively, an intensification of one or other type of feeling—either attachment-related feeling, or a feeling that one is attached—and objectively the sort of behavior likely to result from such intensification. An intensification of either sort of feeling is not an indication of the strengthening of attachment.

This leaves us with the potentially surprising result that, for all the “photograph” and nasal spray studies—and others relevantly like them—are able to show, the action of oxytocin and vasopressin might not be directly correlated with the production or strengthening of human romantic attachment at all. At best, those neurochemicals may simply intensify, so to speak, the trappings of attachment. While it is posited that the data support (A), they in fact support the quite distinct claim (A*). This, it is important to note, is quite a restricted conclusion. It says no more than that the sorts of experimental data often appealed to by the proponents of love drugs are unable to establish what they are taken to establish. It does not follow from this that studies establishing correlation between the action of certain neurochemicals on the one hand and the strengthening of attachment on the other could not be, or indeed have not been, designed. What has been shown is that love-drug enthusiasts need to be cautious about the data they select in order to support their claims, and that the scientists producing those data need to be careful not to confuse feelings of attachment with attachment itself.

## Notes

1. This expression is used in, for example, Fisher H. *Why We Love.* New York, NY: Henry Holt and Company; 2004. Chapter 4. The expression “romantic attraction” is used as the label for the second stage in, for example, Fisher HE , Aron A , Mashek D , Li H , Brown LL . Defining the brain systems of lust, romantic attraction, and attachment. Archives of Sexual Behavior 2002;31(5):413–91223860810.1023/a:1019888024255.

2. See, amongst other publications, Savulescu J , Sandberg A. Neuroenhancement of love and marriage: The chemicals between us. Neuroethics 2008;1:31–44 ; Earp BD , Sandberg A , Savulescu J. Natural selection, childrearing, and the ethics of marriage (and divorce): Building a case for the neuroenhancement of human relationships. Philosophy and Technology 2012;25:561–872322662710.1007/s13347-012-0081-8PMC3510696; Wudarczyk OA , Earp BD , Guastella A , Savulescu J. Could intranasal oxytocin be used to enhance relationships? Research imperatives, clinical policy, and ethical considerations. Current Opinion in Psychiatry 2013;26(5):474–842388059310.1097/YCO.0b013e3283642e10PMC3935449; Earp BD , Sandberg A , Savulescu J. The medicalization of love. Cambridge Quarterly of Healthcare Ethics 2015;24:323–362493763210.1017/S0963180114000206PMC4712680; Earp BD, Savulescu J. *Love is the Drug: The Chemical Future of Our Relationships.* Manchester: Manchester University Press; 2020 (published in North America as *Love Drugs: The Chemical Future of Relationships.* Redwood City, CA: Stanford University Press; 2020). In these publications, the authors count as a love drug any substance that can in principle serve to enhance or rescue a romantic relationship: accordingly, they argue for the use of drugs other than exogenously administered substances, such as oxytocin, that occur endogenously. In their 2020 book, much of their focus falls on the possible use of psychedelics in the service of relationship enhancement/rescue.

3. Earp BD, Savulescu J, Sandberg A. Love drugs and science reporting in the media: Setting the record straight. *Practical Ethics: Ethics in the News* 2012 June 14; available at http://blog.practicalethics.ox.ac.uk/2012/06/should-you-take-ecstasy-to-improve-your-marriage-not-so-fast/ (last accessed 24 Jan. 2022).

4. For an argument against the implied exclusion of attachment from the realm of romantic love, see Jenkins C. What Love Is. New York, NY: Basic Books; 2017, at 22–3.

5. See note 1, Fisher 2004, at 75.

6. See, for example, Young LJ. Love: Neuroscience reveals all. *Nature* 2009;457:148.

7. See note 1, Fisher 2004, at 78.

8. See note 1, Fisher 2004, at 75.

9.

McGee A. Is there such a thing as a love drug? Philosophy, Psychiatry, and Psychology 2016;23(2):79–92 .

10.

Earp BD , Wudarczyk OA , Foddy B , Savulescu J. Addicted to love: What is love addiction and when should it be treated? Philosophy, Psychiatry, and Psychology 2017;24(1):77–92 10.1353/ppp.2017.0011PMC537829228381923. It might be worth noting that this article contains a slight ontological muddle of its own, albeit one not directly relevant to our topic. That is, the authors tell us that “addictions are simply appetites” and that appetites, in turn, are “simply weak addictions” (at 80). They do not appear to notice it follows from this that, although there is a category of weak addictions, there is, implausibly, no category of strong addictions.

11. See note 9, McGee 2016, at 81–2.

12.

Young LJ, Wang Z , Insel TR . Neuroendocrine bases of monogamy. Trends in Neurosciences 1998;21(2):71–5, at 72.949830210.1016/s0166-2236(97)01167-3

13. See note 2, Earp et al. 2015, at 326.

14.

Herissone-Kelly P. Controlling Love: The Ethics and Desirability of Using Love Drugs. Cambridge: Cambridge University Press; forthcoming.

15. See, for example, note 2, Savulescu, Sandberg 2008, at 33, 35, and 37; Earp et al. 2015, at 324.

16. See note 2, Wudarczyk et al. 2013, at 477.

17. See note 2, Wudarczyk et al. 2013, at 479.

18. See note 1, Fisher 2004, at 88.

19. See note 1, Fisher 2004, at 89.

20. See note 1, Fisher 2004, at 89.

21. See note 1, Fisher 2004, at 89.

22. See note 1, Fisher 2004, at 91.

23. See note 1, Fisher 2004, at 93.

